# Silicon reduces impact of plant nitrogen in promoting stalk borer (*Eldana saccharina*) but not sugarcane thrips (*Fulmekiola serrata*) infestations in sugarcane

**DOI:** 10.3389/fpls.2014.00289

**Published:** 2014-06-20

**Authors:** Malcolm G. Keeping, Neil Miles, Chandini Sewpersad

**Affiliations:** ^1^South African Sugarcane Research InstituteMount Edgecombe, South Africa; ^2^School of Animal, Plant, and Environmental Sciences, University of the WitwatersrandJohannesburg, South Africa; ^3^School of Agricultural, Earth and Environmental Sciences, University of KwaZulu-NatalPietermaritzburg, South Africa

**Keywords:** plant nutrition, nitrogen fertilizer, sugarcane cultivars, calcium silicate, integrated pest management, silicon-mediated resistance, thrips, stalk borer

## Abstract

The stalk borer *Eldana saccharina* Walker (Lepidoptera: Pyralidae) is a major limiting factor in South African sugarcane production, while yield is also reduced by sugarcane thrips *Fulmekiola serrata* Kobus (Thysanoptera: Thripidae). Borer management options include appropriate nitrogen (N) and enhanced silicon (Si) nutrition; the effect of N on sugarcane thrips is unknown. We tested the effects of these nutrients, in combination with resistant (N33) and susceptible (N27) sugarcane cultivars, on *E. saccharina* and *F. serrata* infestation. Two pot trials with three levels of N (60, 120, and 180 kg ha^-1^) and two levels each of calcium silicate and dolomitic lime (5 and 10 t ha^-1^) were naturally infested with thrips, then artificially water stressed and infested with borer. Higher N levels increased borer survival and stalk damage, while Si reduced these compared with controls. Silicon significantly reduced stalk damage in N27 but not in N33; hence, Si provided relatively greater protection for susceptible cultivars than for resistant ones. High N treatments were associated with greater thrips numbers, while Si treatments did not significantly influence thrips infestation. The reduction in borer survival and stalk damage by Si application at all N rates indicates that under field conditions, the opportunity exists for optimizing sugarcane yields through maintaining adequate N nutrition, while reducing populations of *E. saccharina* using integrated pest management (IPM) tactics that include improved Si nutrition of the crop and reduced plant water stress. Improved management of N nutrition may also provide an option for thrips IPM. The contrasting effects of Si on stalk borer and thrips indicate that Si-mediated resistance to insect herbivores in sugarcane has mechanical and biochemical components that are well developed in the stalk tissues targeted by *E. saccharina* but poorly developed in the young leaf spindles where *F. serrata* occurs.

## INTRODUCTION

Lepidopteran stalk borers are major pests of sugarcane in almost all regions of the world where this crop is grown (Leslie, 2004). In South Africa, the indigenous pyralid borer *Eldana saccharina* Walker has been the crop’s major pest since the early 1970s after it invaded sugarcane from its natural wetland host plants and gradually spread through the sugar industry (Carnegie, 1974; Atkinson et al., 1981). The direct loss in revenue for cane farmers due to borer activity reducing stalk sucrose content is estimated at US$25,760,000 annum^-1^; however, the indirect costs associated with harvesting the crop when it is only 12 months old (a strategy designed to minimize stalk borer damage), rather than at 15–18 months during its maximum sucrose accumulation period, are more substantial and estimated at US$63,290,000 annum^-1^ (Rutherford, unpublished data). This makes the total annual loss to the industry about US$89,050,000 (Baker, 2014). Nonetheless, early harvesting to avoid the build-up of economically damaging infestations, along with other cultural practices, most important of which is the use of resistant cultivars (Keeping, 2006; Ramburan et al., 2009), remain the most widely used tactics for managing the borer. The insecticide, α-cypermethrin, has also been deployed with noteworthy success to suppress borer populations in cane that is to be aged or “carried over” from one milling season to the next (Leslie, 2009).

Another commonly used cultural control measure has been to reduce applications of nitrogen (N) fertilizer. The effects of N on host plant nutritional quality and herbivorous insect survival and growth have been widely studied (e.g., Mattson, 1980; White, 1984; Coley et al., 2006). In sugarcane, infestations of *E. saccharina* can be exacerbated by high plant N and water stress (Atkinson and Nuss, 1989). Growers were therefore encouraged to reduce their fertilizer applications by 10–30 kg N ha^-1^, depending on the N mineralization potential of the soil, and the likelihood of water stress and borer infestation (Anon, 2005). There is, however, a yield penalty associated with this practice and there is growing recognition that farmers need to revert to recommended rates of N fertilizer that will optimize yields but not incur the risk of heavy borer damage (Stranack and Miles, 2011; Rhodes et al., 2013).

With these factors in mind, integrated pest management (IPM) of *E. saccharina* needs to incorporate plant nutrition practices that render the crop less attractive to or less supportive of the borer, while simultaneously providing beneficial (or at least no detrimental) effects on crop growth and yield (Keeping et al., 2013). Thus, a balance needs to be sought between these two potentially opposing requirements. One way to address this balance is through the enhancement of the crop’s silicon (Si) status. Silicon has historically been neglected in plant nutrition due to its non-essentiality in higher plants (Epstein, 1999), but its fundamental importance in amelioration of abiotic and biotic stresses, is now beyond doubt (Epstein, 2009). Of the biotic stresses that Si can alleviate, insect herbivores and fungal pathogens are especially prominent (Reynolds et al., 2009; Romero et al., 2011); and in Si-accumulating crops such as sugarcane, the capacity of Si to constrain damage by stalk borers, including *E. saccharina*, has now been well documented (Reynolds et al., 2009). There are several mechanisms whereby Si can mediate plant defense against insect herbivores: (1) increased physical (passive) resistance due to amorphous silica deposited in plant tissues, leading to their reduced digestibility and/or increased hardness and abrasiveness (Massey et al., 2006; Massey and Hartley, 2009); (2) active priming of plant chemical defenses by soluble Si and its interaction with the jasmonate (JA) signaling pathway, facilitating enhanced production of defensive enzymes (Gomes et al., 2005; Costa et al., 2011; Ye et al., 2013); (3) indirect defense based on augmented release of herbivore-induced plant volatiles (HIPVs) that attract natural enemies of the attacking herbivore (Kvedaras et al., 2010). In sugarcane, Si-mediated resistance to *E. saccharina* includes physical resistance to stalk penetration by young larvae associated with silica deposits in the stalk epidermis, leading to increased mortality and slower larval growth (Kvedaras and Keeping, 2007; Keeping et al., 2009). However, priming of plant chemical defenses, as in (2) above cannot be excluded.

Most of the earlier work on Si nutrition in sugarcane focused on yield responses (which in many instances were substantial) to application of Si-rich materials to low-Si soils (e.g., Ayres, 1966; Anderson et al., 1991; Berthelsen et al., 2001a). Hence, in South Africa, provision of Si to this crop in regions where its endogenous availability is limited could potentially deliver yield improvements that derive from both its direct effects on plant growth and its indirect effects in suppressing borer damage. The improved (Si-mediated) resistance of the crop to borer may also facilitate a return to recommended rates of N for optimal crop growth. A recent field study by Rhodes et al. (2013) showed that *E. saccharina* responded positively to increasing rates of N in only a minority of cases, supporting the argument that reducing N rates to levels below those required for optimum growth is not warranted.

Support for the idea that Si could be used to offset the promotional effects of N on *E. saccharina* development in sugarcane was first presented by Meyer and Keeping (2005), based on a preliminary potted cane study including five cultivars. Maximum reductions in percent stalk length bored using Si amendment at 200 kg ha^-1^ ranged from 70% at the lowest N level (60 kg ha^-1^), to 39% at the intermediate N level (120 kg ha^-1^) and 35% at the highest N treatment (180 kg ha^-1^). However, their experimental design did not allow for analyses of independent or interactive effects of N and Si. Sétamou et al. (1993) tested the effects of N and Si in separate experiments on the bionomics of *Sesamia calamistis* Hampson (Lepidoptera: Noctuidae) in maize; their findings were consistent with the hypothesis that Si may reduce insect performance (by increasing plant resistance) under conditions of high N fertilization. Hanisch (1981) reported that increasing rates of N and Si augmented and suppressed, respectively, reproduction in *Sitobion avenae* (F.) and *Metopolophium dirhodum* (Walker, Homoptera: Aphididae) on wheat plants, but did so differentially depending on aphid species. Nabity et al. (2012) similarly found that N and Si fertilization of three energy crops differentially affected leaf consumption by two insect herbivores and that consumption depended on herbivore tolerance of high Si diets. Such findings emphasize the likely different outcomes that a plant-nutritional IPM approach could have on different pests (especially from different feeding guilds) attacking the same crop (Keeping and Kvedaras, 2008), and that crop nutrition could possibly be tailored according to pest prevalence in different areas with different soils and climate to achieve optimum yields.

The management of pests in South African sugarcane was further complicated by the appearance of the exotic sugarcane thrips *Fulmekiola serrata* Kobus (Thysanoptera: Thripidae) in 2004 (Way et al., 2006b) and its subsequent establishment throughout the industry. The insect attacks the young leaves emerging at the top of the plant, where its sap-sucking activity causes leaf yellowing, desiccation and binding together of the leaf tips (Williams, 1956; Way et al., 2006a). Measured yield reductions (tons sucrose ha^-1^) of between 16 and 24% (Way et al., 2010) can probably be attributed partly to loss of photosynthetic activity in the damaged leaves. Information on this pest is scant, even from its oriental region of origin, and no conclusive studies have been conducted on its response to plant nutrition. Preliminary work by Keeping et al. (2010), however, indicated that Si provision to potted sugarcane had no effect on the number of thrips recovered from plants. Current control measures include cultivar resistance, manipulation of planting dates to avoid summer thrips population peaks, and systemic insecticides (Keeping et al., 2008; Leslie and Moodley, 2011).

The primary aim of the present study was to investigate the independent (i.e., main) and interactive effects of plant nitrogen and silicon on *E. saccharina* and *F. serrata*, and to establish whether these effects interacted in any way with sugarcane cultivar as a treatment. Previous work has shown that Si has a greater effect in protecting susceptible sugarcane cultivars against borer attack, especially when plants are water stressed (Kvedaras et al., 2007b). The possibility therefore exists that N has a differential effect on resistance of genotypically susceptible and resistant cultivars to one or both pests, as does the possibility that Si differentially affects plant resistance depending on plant N status and the insect herbivore involved. For *F. serrata*, nothing is known of its response to N in any plant. As Si frequently offers little or no benefit to unstressed plants (Epstein, 2009), and because water stress increases susceptibility of sugarcane to *E. saccharina* (Atkinson and Nuss, 1989) but amplifies the effect of Si in protecting against the borer (Kvedaras and Keeping, 2007), we induced water stress *equally* in all plants before borer infestation (see below) to allow clear differentiation of treatment effects.

The results presented here were initially published in condensed form as an un-refereed short communication (Keeping et al., 2012) in the *Proceedings of the Annual Congress of the South Africa Sugar Technologists’ Association* (ISSN 1028-3781).

## MATERIALS AND METHODS

As our study was aimed at testing the principles detailed above, we chose to conduct experiments using potted sugarcane, where extraneous conditions and infestation levels of *E. saccharina* could be better controlled than in field trials. Importantly, we also wished to impose artificial water stress over the period of *E. saccharina* infestation, which necessitated exclusion of rainfall from the plants. The trials were therefore established in a shade house (14 m × 25 m × 3.3 m) with clear polycarbonate roofing and walls of 40% green-shade cloth, at the South African Sugarcane Research Institute (SASRI), Mount Edgecombe, South Africa (29°42′0″ S; 31°2′0″ E), over two successive seasons (early December 2009 and 2010). Planting the trials in summer ensured exposure of the young plants to natural infestation by sugarcane thrips, which preferentially attack young plants (Keeping et al., 2008) and reach a peak in population numbers during December and January in KwaZulu-Natal, South Africa, whereafter numbers drop rapidly going into winter (Way et al., 2007).

### TREATMENTS AND DESIGN

Sugarcane transplants were produced from single-budded setts, cut from mature stalks of two commercial cultivars, one relatively resistant (N33) and the other susceptible (N27) to both *E. saccharina* and *F. serrata*. One-month-old transplants of each cultivar were planted into 25 L PVC pots (three seedlings per pot), with perforated bases, containing 31 kg (dry weight) of clean, sieved and thoroughly leached river sand. Silicon treatments were applied before planting by thoroughly mixing a calcium silicate slag (Calmasil^®^, supplied by PDB Holdings, Pty (Ltd), Middelburg, RSA) into the sand at rates equivalent to 5 t ha^-1^ and 10 t ha^-1^. Calmasil has a Si content of 10.3%, with a neutralizing capacity of 101% of that of calcium carbonate; it also contains 29.5% calcium (Ca) and 6.7% magnesium (Mg). In order to balance for the effect of Calmasil on sand pH, and Ca and Mg supply, controls (with no Si treatment) received equivalent amounts of 5 t ha^-1^ and 10 t ha^-1^ of thoroughly incorporated dolomitic lime, containing 21.0% Ca and 8.1% Mg.

Four pots, containing each of the above four soil treatments, were placed linearly into a total of 48 galvanized metal troughs (200 cm long × 40 cm wide × 10 cm deep). Nutrient solution placed in the troughs supplied the N treatments in the form of ammonium sulfate and di-ammonium phosphate at three different N rates equivalent to 60 kg ha^-1^ (“N1”), 120 kg ha^-1^ (“N2”), and 180 kg ha^-1^ (“N3”). The solution also supplied adequate amounts of potassium, phosphorus, and sulfur (as potassium sulfate, potassium phosphate, and potassium chloride), as well as additional Ca and Mg. Two liters of a 20 L stock solution containing these chemicals were added to 20 L water in each trough and the latter then topped up with 18 L water to a total of 40 L per trough. Every fortnight, the troughs were emptied and fresh nutrient solution supplied as above. Micronutrients (Hygrotech^®^ Micronutrient Hydroponic Seedling Mix) were supplied in solution at a rate of 1.0 g L^-1^ water per pot, added directly to each pot by hand the day after the N solutions were changed. “Skirts” of black plastic sheeting (250 μm thick) were placed over the troughs, with holes cut to fit tightly around the pots, to reduce evaporation and prevent algal growth. Although restriction of root growth in pot experiments is unavoidable, the unlimited supply of water and nutrients during the growth phase (i.e., before water stressing) and the ability of roots to egress into the water troughs through holes in the pots would have assisted in reducing pot binding.

For Trial 1 (2009), the N treatments were introduced 10 weeks after the trial was planted. Until that stage, pure water was supplied via the troughs, while an N:P:K fertilizer was applied to the sand surface once at planting (16 g L^-1^ water per pot) and nutrient seedling mix was supplied fortnightly in solution at 160 g L^-1^ water per pot. For Trial 2 (2010), the N treatments commenced at planting and the seedling mix applied fortnightly as above when the N solutions were renewed.

Pots were arranged in a split-split-plot design with eight replications, where whole plot was “cultivar” (=three troughs in a row), sub-plot was “nitrogen” (=one trough) and sub–sub plot was “silicon” (=one pot). Each row in a trial consisted of three troughs and each trial contained a total of 192 pots. Save for the differences between trials in commencement of N treatments, the treatments and replications were identical; however, the design was re-randomized.

### THRIPS SAMPLING

The trials were naturally infested by *F. serrata* entering the shade houses from the surrounding field-grown sugarcane. At 3 month’s age, all pots in both trials were sampled for *F. serrata* by removing the leaf spindle (i.e., the young fully furled leaves at the apex of the plant) plus adjacent first unfurled leaf from one plant per pot. Spindles plus first leaf were collected into plastic Ziploc bags, which were immediately transferred to a freezer (–24°C) before assessment at a later time. Thrips numbers were assessed by washing the thrips off the spindle with warm water and a few drops of detergent in a plastic tray, and counting their numbers (nymphs plus adults per spindle) under a dissecting microscope. Trial 1 was assessed only for the effects of Si (not N) on thrips numbers in the leaf spindle.

Following the thrips sampling, the trials were sprayed with insecticide monthly (chlorpyriphos 2 mL L^-1^ water) to prevent feral infestations of *E. saccharina* and other pests (aphids, scale, leafhoppers). Spraying was halted 2 months before inoculation with *E. saccharina* (see below) to allow time for pesticide residue on the plants to degrade.

### LEAF AND SOIL SAMPLING

At four month’s age, third leaf (top visible dewlap or TVD) samples for nutrient analysis were taken from each plant in every pot, and leaves from pots with identical treatment combinations were combined between two adjacent replicates. This produced four samples per treatment combination to obtain sufficient leaf material for analysis. Leaf blades were stripped from the midrib and the blades dried, ground, and submitted for N, P, K, Ca, and Si analysis. Trial 1 was also sampled at 10 months.

Soil samples were taken from the trials at 5.5 months to establish responses in levels of soil Ca, Mg, Si, and pH (in 0.01 M calcium chloride) to the Calmasil and lime treatments. Silicon was extracted using 0.02 N sulphuric acid (Kanamugire et al., 2006) for Trial 1 and 0.01 M CaCl_2_ (Berthelsen et al., 2001b; Miles et al., 2011) for Trial 2; Si concentrations were determined using the ammonium molybdate method (Fox et al., 1967). The change in Si extraction method followed routine changes to soil analysis, including improved soil Si determination with CaCl_2_ extraction (Miles et al., 2011).

### WATER STRESSING

At 7 months, the troughs were removed from beneath the pots and the latter placed directly onto the gravel floor of the shade house in their exact original location. Thereafter, the pots were drip irrigated for 15 min daily for one week using 2 L h^-1^ pressure-compensating drippers (=1.0 L pot^-1^ day^-1^). Irrigation was then reduced weekly to 10 min (666 mL pot^-1^), 7 min (466 mL pot^-1^), 5 min (333 mL pot^-1^) and 3 min (200 mL pot^-1^) per day to impose an incremental increase in water stress *across all pots* over 4 weeks (i.e., it did not constitute a treatment). Water stress promotes *E. saccharina* larval survival and development (Atkinson and Nuss, 1989) and ensures a level of infestation sufficient to clearly discriminate treatment effects (Keeping, 2006; Kvedaras et al., 2007b). Care was taken to ensure that a minimum of four green leaves remained on all plants (Inman-Bamber, 2004), by supplying additional water if necessary.

### BORER INFESTATION AND HARVEST

After 4 weeks of increasing water stress (about 8 month’s age), the trials were artificially infested with *E. saccharina*. This entailed careful inoculation of plants with batches of 150 fertilized eggs (laid on small pieces of paper towel) placed behind a lower leaf sheath of one primary tiller in each pot (Keeping, 2006). At the time of inoculation, most of the eggs were in the “black head” stage of development and hatched within 24 h, reducing exposure to ant predation. Surviving larvae boring into the stalks were allowed to develop for 9 weeks in Trial 1 and for 12 weeks in Trial 2. This equated to about 600 degree-days of development, measured using Tempest^®^ Degree-day Units placed in the trials (Insect Investigations Ltd, Cardiff, UK; *t* = 10°C), by which time the majority of larvae were present as late instars (Way, 1995) and the trials could be harvested.

At harvest, stalks from every pot were removed at the base and their length determined. Stalks were then bisected lengthwise to extract and count all surviving larvae and pupae, and to establish the length of stalk bored. The percentage of total stalk length bored was used as a measure of borer damage in the analysis.

### DATA ANALYSIS

All data were tested for univariate normality (Anderson Darling or Shapiro–Wilk tests) and homogeneity of variance (Bartlett’s test) prior to analysis of variance (ANOVA). Where conditions for parametric analysis were not met, appropriate transformations (log or square root) were applied. Where data were unbalanced, as in the case of the leaf Si data for Trial 1 (at 4 months of age), a Residual Maximum Likelihood Ratio (REML) or General Linear Mixed Model (GLMM, with a negative binomial distribution) analysis was performed instead of ANOVA, and only main effects were tested. Probability (*p*) values in the text are derived from these analyses, unless stated otherwise. Where these initial analyses yielded significant differences between treatments, Holm–Sidak *post hoc* tests were applied to determine location of differences.

Effects of cultivar and nitrogen treatments on soil Ca, Mg, Si, and pH are excluded from the results, as the latter were intended only to provide a base-line indication of soil properties of importance to our aims. Similarly, effects of cultivar and N on leaf Ca, Mg, and Si are excluded as these were beyond the scope of the study; analyses revealed that no significant interactions occurred between the main effects.

## RESULTS

### SOIL ANALYSES – TRIAL 1

Calmasil resulted in significantly higher levels of soil Ca, Mg, and Si than lime, although differences in Ca and Mg between the 5 and 10 t ha^-1^ rates of each treatment were non-significant (**Table 1**). Soil Si from the 10 t ha^-1^ Calmasil treatment was 84% higher than that from Calmasil at 5 t ha^-1^ (**Table 1**). There were no significant differences in soil pH between lime and Calmasil treatments, although means for Calmasil were somewhat higher than for lime (**Table 1**).

**Table 1:** Soil analysis for Trials 1 and 2 at 5.5 months in response to lime and Calmasil treatments.

Treatment	Ca (mg kg^-1^)	Mg (mg kg^-1^)	Si (mg kg^-1^)*	pH (CaCl_2_)
**Trial 1**
Lime 5 t ha^-1^	397 ± 30 a	90 ± 4 a	11.0 ± 3.1 a	5.7 ± 0.5
Lime 10 t ha^-1^	442 ± 22 a	99 ± 8 ab	12.0 ± 3.6 a	6.0 ± 0.8
Calmasil 5 t ha^-1^	822 ± 21 b	158 ± 5 bc	50.0 ± 4.6 b	6.8 ± 0.1
Calmasil 10 t ha^-1^	896 ± 86 b	137 ± 16 c	95.7 ± 6.9 c	7.0 ± 0.3
*p* value	<0.001	0.002	<0.001	0.34
**Trial 2**
Lime 5 t ha^-1^	593 ± 45 a	98 ± 15	6.9 ± 0.6 a	7.1 ± 0.1 a
Lime 10 t ha^-1^	868 ± 91 ab	127 ± 28	6.9 ± 0.5 a	7.2 ± 0.1 a
Calmasil 5 t ha^-1^	1019 ± 80 bc	106 ± 20	15.2 ± 0.6 b	7.8 ± 0.1 b
Calmasil 10 t ha^-1^	1277 ± 143 c	99 ± 8	19.9 ± 0.7 c	8.0 ± 0.1 b
*p* value	<0.001	0.66	<0.001	<0.001

Values are means ± standard error (decimal figures are given only where deemed necessary). Probability (*p*) values are from ANOVA. Means within the same column followed by the same letter are not significantly different (Holm–Sidak, *p* < 0.05). *Silicon extracted using H_2_SO_4_ in Trial 1 and CaCl_2_ in Trial 2.

### SOIL ANALYSES – TRIAL 2

Calcium and Mg levels in Trial 2 were similar to Trial 1; however, Si values were substantially lower for the Calmasil treatments in Trial 2 due to the CaCl_2_ extraction method, which gives a more reliable estimate of plant-available Si. There were also no significant differences in soil Mg (**Table 1**). In this instance, soil pH was significantly higher for both rates of Calmasil than for lime, but differences between rates within each treatment were not significant (**Table 1**).

### LEAF ANALYSES – TRIAL 1

Cultivars did not differ in leaf Si content (*p* = 0.7; 2.4 ± 0.2 g kg^-1^ for N27 and N33). However, leaf N was significantly higher in N27 (17.4 ± 0.5 g kg^-1^) than in N33 (15.1 ± 0.3 g kg^-1^; *p* < 0.001). Leaf N increased significantly (*p* < 0.001) between all N treatment rates from 14.2 ± 0.3 g kg^-1^ (N1), through 16.1 ± 0.5 g kg^-1^ (N2), to 18.4 ± 0.5 g kg^-1^ (N3; Holm–Sidak, *p* < 0.05).

Leaf Ca and Si were both significantly higher in the Calmasil than in the lime treatments; leaf Si in the 10 t ha^-1^ Calmasil treatment was also significantly (42%) higher than Calmasil at 5 t ha^-1^ (**Table 2**). There was no significant effect of the Calmasil and lime treatments on leaf Mg content (**Table 2**).

**Table 2:** Leaf analysis for Trials 1 and 2 at 4 months (Ca, Mg, Si) and 10 months (Si only) in response to lime and Calmasil treatments.

Treatment/statistic	Ca (g kg^-1^)	Mg (g kg^-1^)	Si (g kg^-1^) 4 months	Si (g kg^-1^) 10 months
**Trial 1**
Lime 5 t ha^-1^	1.8 ± 0.2 a	1.0 ± 0.03	1.7 ± 0.2 a	–
Lime 10 t ha^-1^	1.9 ± 0.2 a	0.9 ± 0.04	1.6 ± 0.2 a	–
Calmasil 5 t ha^-1^	2.5 ± 0.3 b	1.0 ± 0.03	2.6 ± 0.3 b	–
Calmasil 10 t ha^-1^	2.7 ± 0.3 b	1.0 ± 0.03	3.7 ± 0.3 c	–
*p* value	0.009	0.24	<0.001	–
**Trial 2**
Lime 5 t ha^-1^	2.8 ± 0.1	1.2 ± 0.03	2.7 ± 0.2 a	1.9 ± 0.1 a
Lime 10 t ha^-1^	2.9 ± 0.1	1.2 ± 0.04	2.6 ± 0.1 a	1.8 ± 0.5 a
Calmasil 5 t ha^-1^	3.1 ± 0.1	1.3 ± 0.05	3.8 ± 0.1 b	4.1 ± 0.2 ab
Calmasil 10 t ha^-1^	2.9 ± 0.1	1.2 ± 0.04	4.3 ± 0.2 b	5.8 ± 1.1 b
*p* value	0.06	0.07	<0.001	0.004

*Values are means ± standard error. Probability (*p*) values are from ANOVA. Means within the same column followed by the same letter are not significantly different (Holm–Sidak, *p* < 0.05).*

### LEAF ANALYSES – TRIAL 2

There was no effect of cultivar on leaf Si (*p* = 0.95; 3.4 ± 0.3 g kg^-1^ for N27 and N33) or on leaf N (*p* = 0.34; N27: 15.7 ± 0.2 g kg^-1^, N33: 15.2 ± 0.2 g kg^-1^). Leaf N did not respond to the N treatments in either the 4-month (p = 0.97) or 10-month (*p* = 0.08) samples. However, at 10 months mean leaf N concentration increased from 9.7 ± 0.8 g kg^-1^ (N1), through 10.4 ± 0.3 g kg^-1^ (N2), to 12.1 ± 0.5 g kg^-1^ (N3).

Leaf Ca and Mg content were not affected by Calmasil and lime treatments (**Table 2**). At 4 months, Calmasil produced significantly higher leaf Si than lime, but the 5 and 10 t ha^-1^ rates did not differ significantly within treatments (**Table 2**). Similar results were recorded at 10 months. Notably, leaf Si content increased from 4 to 10 months in the Calmasil treatments but decreased in the lime treatments (**Table 2**).

### THRIPS INFESTATION

In both trials, cultivar N33 had significantly fewer thrips (*F. serrata*) than N27 (**Table 3**); numbers on N33 were 19% lower in Trial 1 and 32% lower in Trial 2. In Trial 2, the N2 and N3 treatments significantly increased thrips number per spindle over N1 (**Table 3**). The soil treatments (and therefore Si) had no significant effects on thrips abundance (**Table 3**), and there were no significant interactions.

**Table 3:** Thrips abundance in Trials 1 and 2 at 3 months in response to cultivar, soil, and nitrogen treatments.

	Total thrips spindle^-1^	
Treatment/statistic	Trial 1	Trial 2
**Cultivar**
N27	32.2 ± 2.6	22.3 ± 1.5
N33	26.2 ± 1.9	15.2 ± 1.4
*p* value	0.03	0.01
**Nitrogen**
60 kg ha^-1^ (N1)	–	14.13 ± 1.6 a
120 kg ha^-1^ (N2)	–	20.36 ± 1.6 b
180 kg ha^-1^ (N3)	–	21.77 ± 2.2 b
*p* value	–	0.002
**Soil**
Lime 5 t ha^-1^	31.2 ± 3.7	21.94 ± 2.7
Lime 10 t ha^-1^	29.3 ± 3.2	18.56 ± 1.9
Calmasil 5 t ha^-1^	30.2 ± 3.3	16.25 ± 1.6
Calmasil 10 t ha^-1^	26.3 ± 2.6	18.25 ± 2.2
*p* value	0.51	0.23

*Values are means ± standard error. Values for N treatments in Trial 1 are not provided for reasons given in text. Probability (*p*) values are from ANOVA. Means within the same column followed by the same letter are not significantly different (Holm–Sidak, *p* < 0.05).*

### BORER INFESTATION – TRIAL 1

**FIGURE 1:**
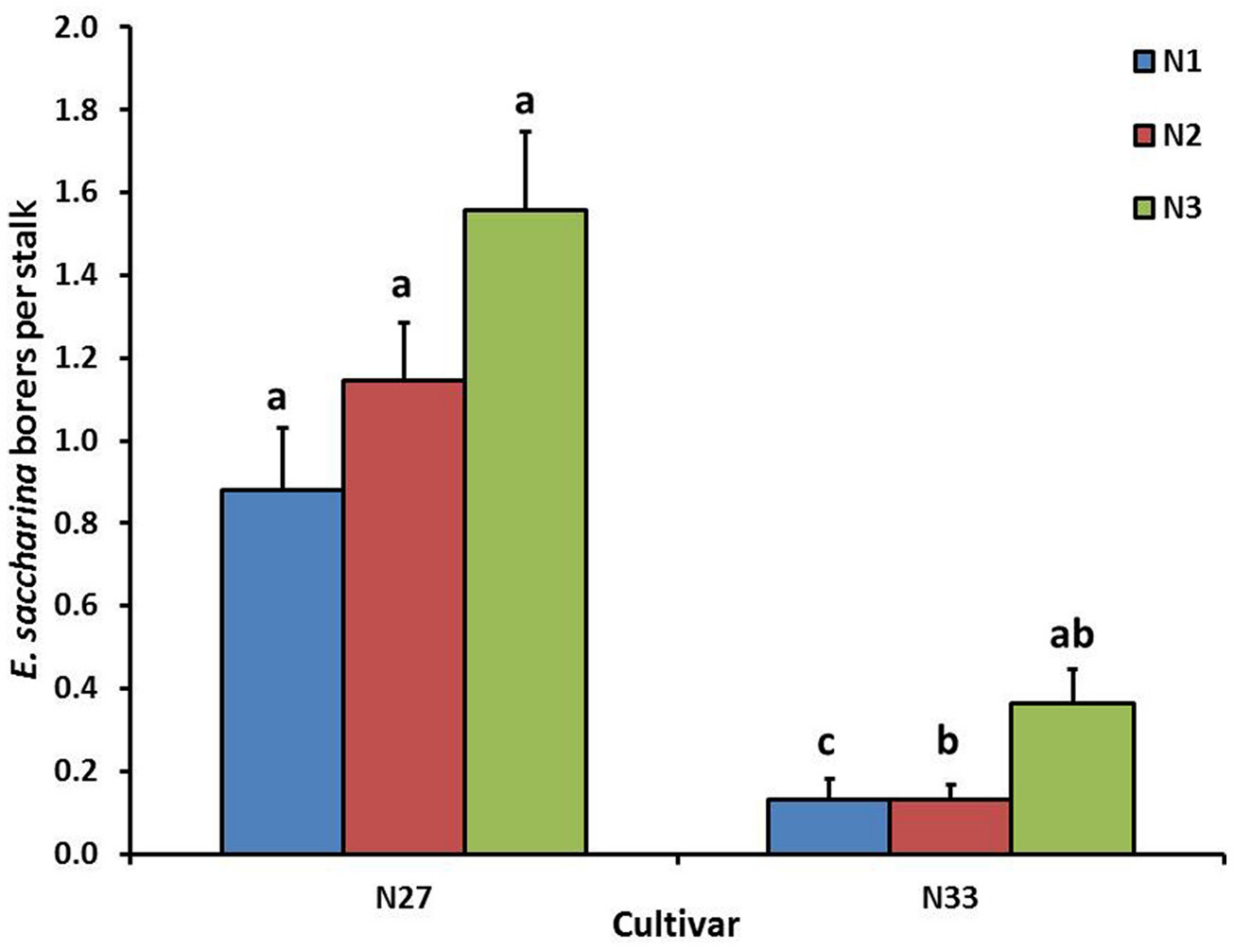
**Mean (±SE) number of *E. saccharina* recovered per stalk from sugarcane cultivars (N27 and N33) fertilized at different N rates (N1, N2, and N3) in Trial 1.** Bars with the same letter/s above them do not differ significantly (Holm–Sidak test, *p* < 0.05).

**FIGURE 2:**
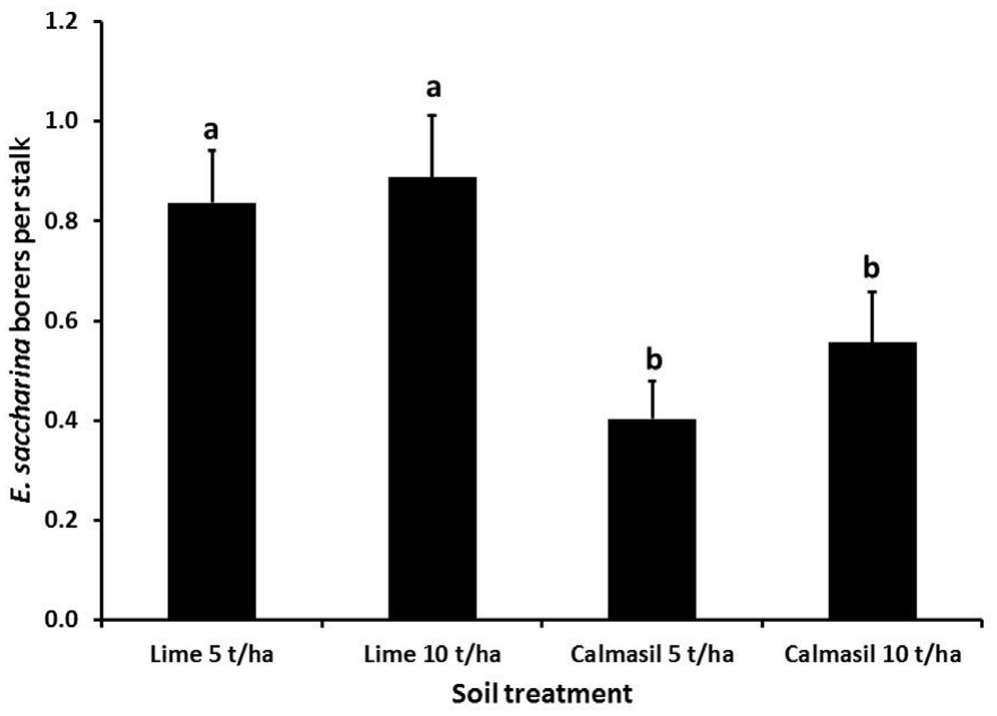
**Mean (±SE) number of *E. saccharina* recovered per stalk from sugarcane amended with Calmasil and lime at two different rates in Trial 1.** Bars with the same letter/s above them do not differ significantly (Holm–Sidak test, *p* < 0.05).

Cultivar significantly affected number of borers (*E. saccharina*) recovered (*p* < 0.001), being 83% lower in N33 (0.2 ± 0.03 borers stalk^-1^) than in N27 (1.2 ± 0.09 borers stalk^-1^). There was a significant interaction between cultivar and nitrogen (*p* = 0.03); although N did not significantly increase borer numbers in N27, there was a significant increase in N33 between N1 and N3, to the extent that the N33 + N3 treatment did not differ significantly from N27 (**Figure 1**). Soil treatments significantly affected borer numbers (*p* = 0.018), which were reduced by an overall 44% in the Calmasil treatments compared with lime (**Figure 2**).

**FIGURE 3:**
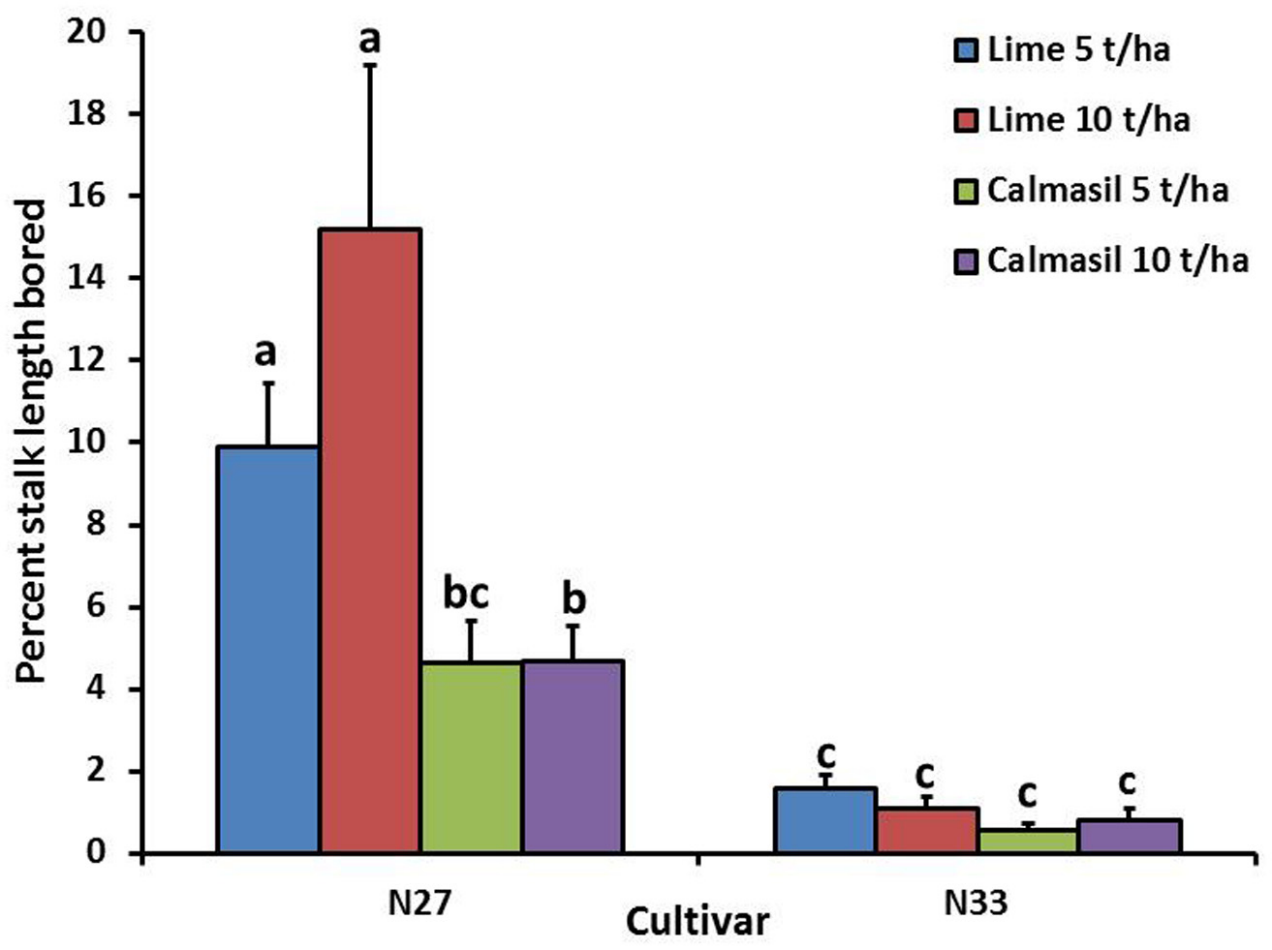
**Mean (±SE) percent stalk length bored by *E. saccharina* in sugarcane cultivars (N27 and N33) amended with Calmasil and lime at two different rates in Trial 1.** Bars with the same letter/s above them do not differ significantly (Holm–Sidak test, *p* < 0.05).

Percent stalk length damaged was significantly reduced (*p* < 0.001) by 88% in N33 (1.0 ± 0.1%) compared with N27 (8.7 ± 1.2%). Nitrogen treatments significantly affected percent stalk length bored (*p* = 0.008), the latter increasing from 2.8 ± 0.5% in N1, through 4.6 ± 1.2% in N2, to 6.1 ± 1.0% in N3; the difference between N1 and N3 was significant (Holm–Sidak, *p* < 0.05). There was a significant interaction between cultivar and soil treatment (*p* = 0.003), wherein the Calmasil treatments had no effect on borer damage in N33, but significantly reduced damage in N27; notably, damage in N27 with Calmasil 5 t ha^-1^ did not differ significantly from that in N33 with lime 5 t ha^-1^ (**Figure 3**).

### BORER INFESTATION – TRIAL 2

**FIGURE 4:**
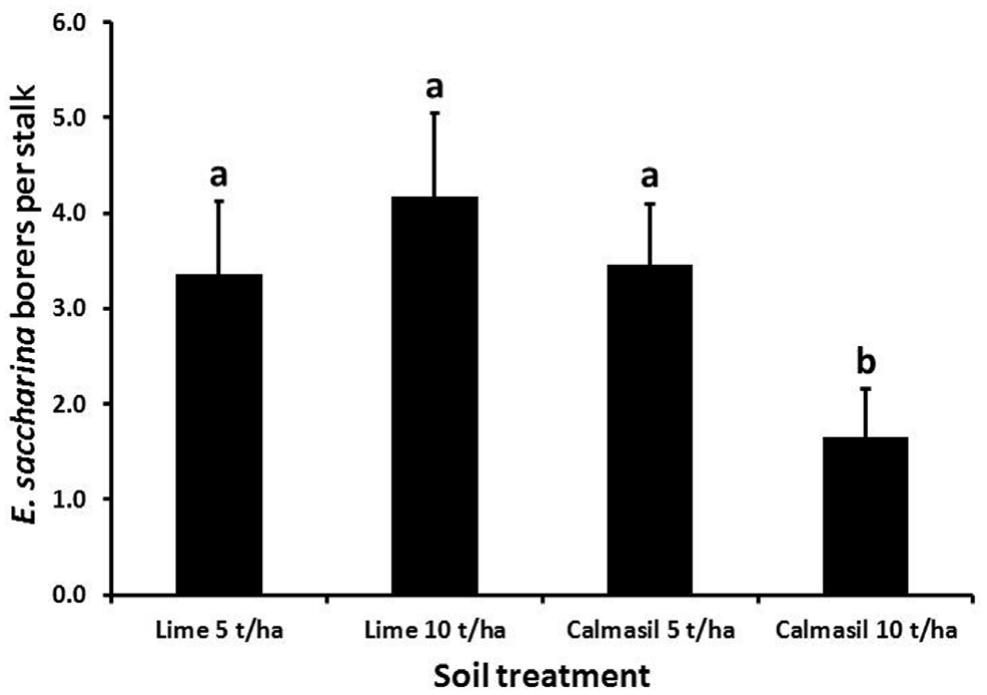
**Mean (±SE) number of *E. saccharina* recovered per stalk from sugarcane amended with Calmasil and lime at two different rates in Trial 2.** Bars with the same letter/s above them do not differ significantly (Holm–Sidak test, *p* < 0.05).

Borer numbers were significantly reduced (by 63%; *p* < 0.001) from 4.6 ± 0.6 borers stalk^-1^ in N27 to 1.7 ± 0.3 borers stalk^-1^ in N33. Nitrogen treatment significantly affected borer recovery (*p* < 0.001), with numbers per stalk increasing from 1.6 ± 0.4 in N1, through 2.9 ± 0.5 in N2, to 4.8 ± 0.8 in N3. Numbers in both N2 and N3 differed significantly from N1 (Holm–Sidak, *p* < 0.05), but not from one another. Soil treatments had a significant effect on borer numbers (*p* < 0.001), wherein the 10 t ha^-1^ Calmasil treatment reduced numbers significantly compared with all other treatments and by 41% compared with 10 t ha^-1^ lime; Calmasil at 5 t ha^-1^ did not reduce numbers compared with lime controls (**Figure 4**). There were no significant interactions between treatments.

**FIGURE 5:**
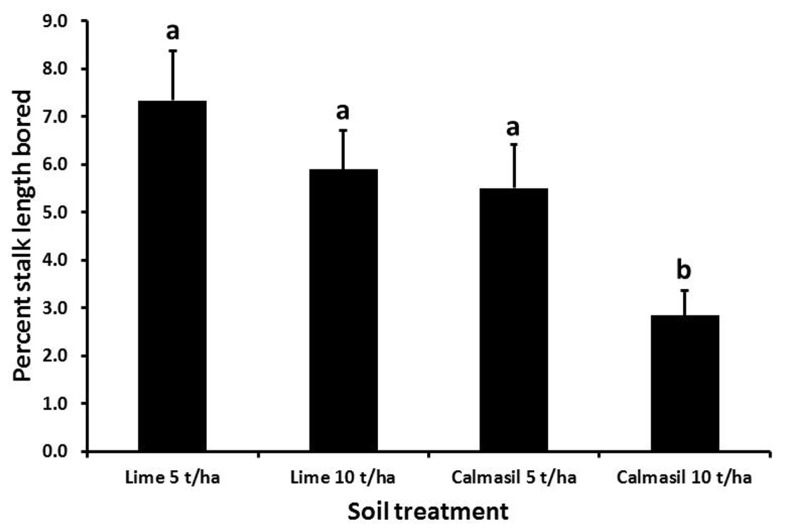
**Mean (±SE) percent stalk length bored by *E. saccharina* in sugarcane amended with Calmasil and lime at two different rates in Trial 2.** Bars with the same letter/s above them do not differ significantly (Holm–Sidak test, *p* < 0.05).

Percent stalk length damaged was significantly reduced (by 54%; *p* < 0.001) from 7.4 ± 0.8% in N27 to 3.4 ± 0.4% in N33. Nitrogen treatments significantly affected percent stalk length bored (*p* < 0.001), the latter increasing from 3.5 ± 0.5% in N1, through 5.5 ± 0.7% in N2, to 7.2 ± 0.9% in N3; both N2 and N3 differed significantly from N1, but not from one another (Holm–Sidak, *p* < 0.05). Borer damage was significantly affected by the soil treatments (*p* < 0.001), with percent stalk length bored reduced significantly in the 10 t ha^-1^ Calmasil treatment compared with all other treatments and by 49% compared with 5 t ha^-1^ lime; Calmasil at 5 t ha^-1^ did not reduce numbers compared with lime controls (**Figure 5**). There were no significant interactions between treatments.

## DISCUSSION

Soil and leaf analyses from both trials conducted in this study demonstrated that Calmasil significantly and consistently raised soil and leaf Si content. There was also a clear positive effect of rate of application on soil and plant Si (**Tables 1** and **2**). While soil Ca and Mg levels were increased more by Calmasil than by dolomitic lime applied at the same rates (**Table 1**), the effects on leaf content of these elements were evident only for Ca in Trial 1 (**Table 2**). The most consistent and largest increases in leaf concentrations of elements provided by the Calmasil treatments were those of Si (**Table 2**). Furthermore, the leaf concentrations of Ca and Mg were within the “satisfactory” range of 1.5–3.9 g kg^-1^ and 0.8–1.9 g kg^-1^, respectively, for South African sugarcane (Miles and Rhodes, 2013). It is unlikely, therefore, that Ca or Mg contributed to the significant effects of the Calmasil treatment on plant resistance to borer reported in this study and discussed below.

Of interest, the cultivars used in this study did not differ in Si content. Previous studies that included the resistant cultivar N33 found that its endogenous Si content was high compared with borer-susceptible cultivars such as N11 and N26 (Kvedaras et al., 2007a; Keeping et al., 2009). Cultivar differences in Si content are also well documented from other countries (e.g., Deren et al., 1993). Since cultivar differences in leaf N content were evident only in Trial 1, it is unlikely that borer responses to cultivar or to N treatments were linked to endogenous differences in cultivar N content.

Although Calmasil significantly increased soil pH above that produced by lime in Trial 2, this did not occur in Trial 1 (**Table 1**), making it unlikely that pH had an effect on leaf Si content and borer resistance. Furthermore, studies conducted in the SA sugar industry indicate that soil pH values in the range recorded here for the lime and Calmasil treatments (i.e., ≥5.5) are generally associated with satisfactory leaf Si concentrations above 10 g kg^-1^ (Van Der Laan and Miles, 2010). The pH values for the Calmasil treatments support the argument that calcium silicate may provide liming capabilities similar or superior to those of dolomitic lime, while providing ample quantities of Ca and Mg (Marafon and Endres, 2013).

There was no evidence that elevated leaf Si mitigated sugarcane thrips infestation, in agreement with the preliminary results of Keeping et al. (2010). While some studies have shown an increase in resistance to various thrips species in response to root-applied silicate treatments (Subramanian and Gopalaswamy, 1988; Almeida et al., 2008), a recent study on *Scirtothrips dorsalis* Hood (Thysanoptera: Thripidae) on pepper plants found minimal effects of Si on damage and thrips numbers recovered from infested plants (Dogramaci et al., 2013). The authors contended that this was due to inadequate accumulation of Si in the epidermis and mesophyll of the young leaves targeted by the insect. In sugarcane, Si content can increase from 4 g kg^-1^ in young leaves to more than 60 g kg^-1^ in old ones (Van Dillewijn, 1952). Analysis of Si content of TVD leaves and leaf spindles collected from the same tillers of 8-month-old field-grown cane (Keeping, unpublished data), showed significantly lower quantities (*p* < 0.001) of Si in the young spindle tissue (8.4 ± 0.5 g kg^-1^) than in the TVD (15.3 ± 1.4 g kg^-1^). We argue here that, as for *S. dorsalis* on pepper plants, the accumulated amorphous Si in the leaf spindle of Calmasil treated cane was insufficient to provide a suppressive effect on *F. serrata* populations and therefore unlikely to be of value in crop protection against this pest. It may also partly explain why the leaf spindle is the favored microhabitat for sugarcane thrips, together with the more protected environment and softer foliage that the spindle offers.

Inert, amorphous silica (plant opal) presents insect herbivores with mechanical difficulties in chewing, penetrating, and digesting plant tissues (Massey et al., 2006; Massey and Hartley, 2009; Reynolds et al., 2009). However, soluble Si appears also to be involved as plants attacked by insects and simultaneously fed with Si may show enhanced expression of several defensive enzymes compared with Si-untreated plants (Gomes et al., 2005). Most recently, Ye et al. (2013) showed that Si amendment amplified the JA mediated defense responses of rice to *Cnaphalocrocis medinalis* (Guenée, Lepidoptera: Pyralidae), and therefore served as a priming agent in this crucial plant defense signaling pathway. Although the JA pathway is active in sugarcane (Bower et al., 2001), monthly applications of JA to the leaves of Si-amended potted sugarcane failed to suppress *F. serrata* populations (Keeping et al., 2010), indicating that the effects of both Si and JA in eliciting biochemical defenses in young sugarcane tissue are indistinguishable.

Fertilization with N at 120 and 180 kg ha^-1^ significantly increased thrips recovery from leaf spindles, compared with the N rate of 60 kg ha^-1^. While there is abundant evidence for the promotional effects of higher N rates on populations of other thrips, especially* Frankliniella* spp. (fam. Thripidae, e.g., Brodbeck et al., 2001; Chau et al., 2005; Atakan, 2006; Baez et al., 2011), our study is the first to report on effects of N on *F. serrata*. In sugarcane, the concentrations of N, Si, potassium (K), and phosphorus (P) show a marked decrease during the first three to four months of growth; furthermore, concentrations of N and K decrease considerably in individual leaves as they age (Van Dillewijn, 1952). The initially high levels of N in young plants and in young leaves may partly explain why *F. serrata* populations are substantially higher in young plants (1–3 months old) and why they favor the leaf spindle. Avoidance of N application at higher than recommended rates for optimal cane growth is therefore an important management requirement for sugarcane thrips. This can be integrated with other practices, such as planting well before or after the mid-summer population peaks to prevent exposure of young plants to high infestation pressure (Keeping et al., 2008), in combination with more resistant cultivars (this study, Keeping et al., 2008; Joshi et al., 2014) and carefully timed insecticide applications (Leslie and Moodley, 2011).

Our study confirms that resistant cultivars contribute substantially to managing infestations of both pests: compared to N27, *F. serrata* numbers were reduced in N33 by 19 and 32%, and *E. saccharina* numbers by 63 and 83% in Trials 1 and 2, respectively; borer damage was reduced by 54 and 88%. Although no quantitative index has been developed between yield loss and *F. serrata* numbers or leaf injury, reduced thrips numbers are associated with increased sucrose yields (Way et al., 2010), while the suppression of borer damage produces an equivalent saving in sucrose based on an established (but probably conservative) 1% sucrose lost for every 1% internodes bored (King, 1989). The existence of cultivars such as N33, with cross-resistance to both pests, is fortunate for both growers and plant breeders; the latter, as it can serve as a parent in crosses aimed at improving pest resistance in the sugarcane gene pool.

Higher rates of N fertilization and resultant higher plant N significantly increased *E. saccharina* survival (i.e., recovery) and damage in both trials, in concurrence with several pot and field studies of this borer (Carnegie, 1981; Atkinson and Nuss, 1989; Coulibaly, 1990; Meyer and Keeping, 2005; Van Antwerpen et al., 2011). Plant water stress is an additional factor that increases the mobilization and availability of N to herbivores (White, 1984), including *E. saccharina* (Atkinson and Nuss, 1989), and enhances insect performance in the majority of stem borers (Galway et al., 2004; Huberty and Denno, 2004). Furthermore, the significant interaction between cultivar and N in Trial 1 (**Figure 1**) suggests that the promotional effects of N may be such that a resistant cultivar that is simultaneously water stressed and fertilized at high N rates may be rendered as suitable for borer growth as a susceptible (water stressed) cultivar fertilized at low N rates. Similarly, in the absence of different N fertilization rates, water-stressed resistant cultivars may have similar borer-susceptibility to non-stressed, susceptible cultivars (Kvedaras et al., 2007b).

Notwithstanding these observations, several field studies and surveys from commercial cane where water stress was not controlled have found no clear relationship between N rate and *E. saccharina* infestation (Carnegie, 1981; Atkinson and Nuss, 1989; Stranack and Miles, 2011; Rhodes et al., 2013). In 16 harvested crops from 10 field trials, Rhodes et al. (2013) recorded significant increases in infestation in response to N in only three crops, despite seven of the crops growing over a severe drought period during 2010. A reduction in N rate for each trial to 20 kg ha^-1^ less than the recommended rates would not have achieved significant reductions in borer damage (Rhodes et al., 2013). Cultivar and its interaction with N (this study) and water stress (Kvedaras et al., 2007b) almost certainly play an important role in producing such variable responses to borer under field conditions. Most borer-resistant cultivars are also comparatively drought tolerant (Keeping and Rutherford, 2004); as the cultivars used in 7 of the 10 trials conducted by Rhodes et al. (2013) exhibit these characteristics, this, together with soil type, may have affected the impact of higher N rates and water stress on borer infestation.

In contrast to N, Si application significantly reduced borer survival (**Figures 2** and **4**) and damage (**Figures 3** and **5**) in both trials. In Trial 1, the effect of Si on borer damage was contingent on cultivar, with damage significantly diminished in susceptible N27, but not in resistant N33 (**Figure 3**). A greater benefit from Si amendment of susceptible cultivars has previously been reported for *E. saccharina* (Keeping and Meyer, 2002, 2006; Kvedaras et al., 2007b) and *Mahanarva fimbriolata* Stål (Hemiptera: Cercopidae; Korndörfer et al., 2011) in sugarcane, and for *Chilo suppressalis* (Walker, Lepidoptera: Crambidae, Hou and Han, 2010) in rice. The phenomenon has also been widely reported in studies of Si-mediated resistance to plant pathogens, where Si applications have enhanced resistance of susceptible cultivars to levels similar to those of resistant ones (e.g., Datnoff et al., 2001; Rodrigues et al., 2001; Kanto et al., 2006; Fortunato et al., 2012; Shetty et al., 2012). While the cultivars used in this study did not differ in leaf Si content, earlier studies have shown that N33, treated or untreated with Si, displayed higher plant Si content than borer-susceptible cultivars, indicating that some of its resistance is attributable to endogenously higher total plant Si content (Kvedaras and Keeping, 2007; Kvedaras et al., 2007a; Keeping et al., 2009). However, the absence of a difference in total leaf Si content (which is highly correlated with stalk Si%; Keeping, unpublished data) between N33 and N27, yet significantly different responses to Si treatment, indicate that a Si-mediated resistance mechanism is at work over and above a passive silica-based mechanical barrier. We suggest that, as has been demonstrated for a sap-sucker, *Schizaphis graminum* (Rond., Hemiptera: Aphididae, Gomes et al., 2005), on wheat and a leaf-feeder, *C. medinalis* (Ye et al., 2013), on rice, insect-inducible chemical defenses primed by soluble Si may be involved in Si-mediated resistance to *E. saccharina* in sugarcane. Confirmation of this and the possible involvement of the JA pathway would extend the existence of this mechanism to a third, insect-feeding guild (stalk borers).

The high endogenous Si content of N33 (compared with susceptible cultivars like N11 and N26) together with other resistance mechanisms to *E. saccharina*, including high fiber content and stalk rind hardness (Keeping et al., 2009; Kvedaras et al., 2009), likely account for the insignificant effects of Si fertilization (**Figure 3**) on total resistance of N33 to the borer. Such observations highlight that there is little (or no) justification, even at high rates of N, for Si fertilization of resistant cultivars purely for enhancement of stalk borer resistance. However, there are soil health related reasons, in particular wide-spread deficiencies in plant-available Si, and amelioration of soil acidity and aluminum toxicity, that argue persuasively for the continued use of Si-rich liming materials in the rainfed regions of the South African sugar industry (Moberly and Meyer, 1975; Van Der Laan and Miles, 2010; Keeping et al., 2013). Beyond this, there are other benefits, including yield enhancement and improved tolerance of various abiotic stresses, especially drought (Alvarez and Datnoff, 2001), which could justify its use.

In N27, the reduction in borer survival and stalk damage by Si application at all N rates indicates that for more susceptible cultivars planted in rainfed regions, the opportunity exists for optimizing sugarcane yields through maintaining adequate N nutrition, while reducing populations of *E. saccharina* through improved Si nutrition of the crop. However, we emphasize that Si provision would constitute only one component of an IPM strategy that must embrace other available measures (Rutherford and Conlong, 2010), including reduced plant water stress. Previous studies have shown that Si is especially beneficial in water stressed cane (Kvedaras et al., 2007b), but also that the combination of water stress with high N rates provides conditions that support the development of damaging borer infestations (Atkinson and Nuss, 1989). Results from the present study are consistent with both these observations. Excessive applications of N under conditions where lower cane yields are expected are in any case uneconomic and environmentally harmful, and should therefore be avoided (Meyer et al., 2007). We argue here that attention by growers to addressing problems of Si and other nutrient deficiencies, including N and K (see Rhodes et al., 2013 for the latter), is a first step in avoiding plant stress and reducing borer infestation. A second step is to improve soil health and root growth – and hence nutrient and water uptake – by reducing soil acidity and aluminum toxicity through liming and/or calcium silicate provision (Moberly and Meyer, 1975; Meyer et al., 1998; Bell et al., 2002; Van Der Laan and Miles, 2010; Keeping et al., 2013). A third step is to augment and preserve soil moisture through practices that increase soil organic matter and improve rainwater infiltration (Thorburn et al., 1999; Bell et al., 2001; Pankhurst et al., 2005). Such measures, among others, will enable growers to minimize crop stress and re-adopt recommended N fertilization rates without the risk of economic losses to *E. saccharina* infestation.

## AUTHOR CONTRIBUTIONS

Malcolm G. Keeping conceived and conducted the research, with advice and guidance on all plant nutritional aspects from Neil Miles. Trial designs and statistical analyses were performed by Chandini Sewpersad. The manuscript was written by Malcolm G. Keeping, and critiqued and approved by Neil Miles and Chandini Sewpersad.

## Conflict of Interest Statement

The authors declare that the research was conducted in the absence of any commercial or financial relationships that could be construed as a potential conflict of interest.

## References

[B1] Almeida G. D. Pratissoli D. Zanuncio J. C. Vicentini V. B. Holtz A. M. Serrão J. E. (2008). Calcium silicate and organic mineral fertilizer applications reduce phytophagy by *Thrips palmi* Karny (Thysanoptera: Thripidae) on eggplants (*Solanum melongena* L.). *Interciencia* 33 835–838

[B2] Alvarez J. Datnoff L. E. (2001). “The economics of silicon for integrated management and sustainable production of rice and sugarcane,” in *Silicon in Agriculture* eds Datnoff L. E. Snyder G. H. Korndörfer G. H. (Amsterdam: Elsevier) 221–239

[B3] Anderson D. L. Snyder G. H. Martin F. G. (1991). Multi-year response of sugarcane to calcium silicate slag on Everglades Histosols. *Agron. J.* 83 870–874 10.2134/agronj1991.00021962008300050019x

[B4] Anon. (2005). *Guidelines and Recommendations for Eldana Control in the South African Sugar Industry*. Mount Edgecombe: South African Sugarcane Research Institute

[B5] Atakan E. (2006). Effect of nitrogen fertilization on population development of *Frankliniella* sp. (Thysanoptera: Thripidae) in cotton in Eastern Mediterranean region of Turkey. *J. Biol. Sci.* 6 868–874 10.3923/jbs.2006.868.874

[B6] Atkinson P. R. Carnegie A. J. M. Smaill R. J. (1981). A history of the outbreaks of *Eldana saccharina* Walker, in Natal. *Proc. S. Afr. Sug. Technol. Ass.* 55 111–115

[B7] Atkinson P. R. Nuss K. J. (1989). Associations between host–plant nitrogen and infestations of the sugarcane borer, *Eldana saccharina* Walker (Lepidoptera: Pyralidae). *Bull. Ent. Res.* 79 489–506 10.1017/S0007485300018460

[B8] Ayres A. S. (1966). Calcium silicate slag as a growth stimulant for sugarcane on low-silicon soils. *Soil Sci.* 101 216–227 10.1097/00010694-196603000-00009

[B9] Baez I. Reitz S. R. Funderburk J. E. Olson S. M. (2011). Variation within and between *Frankliniella* thrips species in host plant utilization. *J. Insect Sci.* 11 41 10.1673/031.011.0141 PMC3281448 21539418

[B10] Baker C. (2014). Getting to grips with Eldana. *The Link* 23 4–5

[B11] Bell M. J. Garside A. L. Moody P. W. Pankhurst C. Halpin N. V. Berthelsen J. (2002). Nutrient dynamics and root health in sugarcane soils. *Proc. Aust. Soc. Sugar Cane Technol.* 24 92–98

[B12] Bell M. J. Halpin N. V. Orange D. N. Haines M. (2001). Effect of compaction and trash blanketing on rainfall infiltration in sugarcane soils. *Proc. Aust. Soc. Sugar Cane Technol.* 23 161–167

[B13] Berthelsen S. Hurney A. Kingston G. Rudd A. Garside A. L. Noble A. D. (2001a). Plant cane responses to silicated products in the Mossman, Innisfail and Bundaberg districts. *Proc. Aust. Soc. Sugar Cane Technol.* 23 297–303

[B14] Berthelsen S. Hurney A. Noble A. D. Rudd A. Garside A. L. Henderson A. (2001b). An assessment of current silicon status of sugar cane production soils from Tully to Mossman. *Proc. Aust. Soc. Sugar Cane Technol.* 23 289–296

[B15] Bower N. I. Casu R. E. Maclean D. J. Manners J. M. (2001). Identification of markers of the defence response in sugarcane. *Proc. Int. Soc. Sug. Technol.* 24 624–625

[B16] Brodbeck B. V. Stavisky J. Funderburk J. E. Andersen P. C. Olson S. M. (2001). Flower nitrogen status and populations of *Frankliniella occidentalis* feeding on *Lycopersicon esculentum*. *Entomol. Exp. Appl.* 99 165–172 10.1046/j.1570-7458.2001.00814.x

[B17] Carnegie A. J. M. (1974). A recrudescence of the borer *Eldana saccharina* Walker (Lepidoptera: Pyralidae). *Proc. S. Afr. Sug. Technol. Ass.* 48 107–110

[B18] Carnegie A. J. M. (1981). Combating *Eldana saccharina* (Lepidoptera: Pyralidae): a progress report. *Proc. S. Afr. Sug. Technol. Ass.* 55 116–119

[B19] Chau A. Heinz K. M. Davies F. T. Jr (2005). Influences of fertilization on population abundance, distribution, and control of *Frankliniella occidentalis* on chrysanthemum. *Entomol. Exp. Appl.* 117 27–39 10.1111/j.1570-7458.2005.00326.x

[B20] Coley P. D. Bateman M. L. Kursar T. A. (2006). The effects of plant quality on caterpillar growth and defense against natural enemies. *Oikos* 115 219–228 10.1111/j.2006.0030-1299.14928.x

[B21] Costa R. R. Moraes J. C. DaCosta R. R. (2011). Feeding behaviour of the greenbug Schizaphis graminum on wheat plants treated with imidacloprid and/or silicon. *J. Appl. Entomol.* 135 115–120 10.1111/j.1439-0418.2010.01526.x

[B22] Coulibaly K. (1990). Influence of nitrogen fertilization on the attack on sugar cane by the stalk borer (*Eldana saccharina* Walker). *Sugar Cane* Spring Suppl. 18–20

[B23] Datnoff L. E. Seebold K. W. Correa-Victoria F. J. (2001). “The use of silicon for integrated disease management: reducing fungicide applications and enhancing host plant resistance,” in *Silicon in Agriculture* eds Datnoff L. E. Snyder G. H. Korndörfer G. H. (Amsterdam: Elsevier) 171–184

[B24] Deren C. W. Glaz B. Snyder G. H. (1993). Leaf-tissue silicon content of sugarcane genotypes grown in Everglades histosols. *J. Plant Nutr.* 16 2273–2280 10.1080/01904169309364685

[B25] Dogramaci M. Arthurs S. P. Chen J. J. Osborne L. (2013). Silicon applications have minimal effects on *Scirtothrips dorsalis* (Thysanoptera: Thripidae) populations on pepper plant, *Capsicum annum* L. *Fla Entomol.* 96 48–54 10.1653/024.096.0106

[B26] Epstein E. (1999). Silicon. *Annu. Rev. Plant Phys.* 50 641–664 10.1146/annurev.arplant.50.1.641 15012222

[B27] Epstein E. (2009). Silicon: its manifold roles in plants. *Ann. Appl. Biol.* 155 155–160 10.1111/j.1744-7348.2009.00343.x

[B28] Fortunato A. A. Rodrigues F. Á. Baroni J. C. P. Soares G. C. B. Rodriguez M. A. D. Pereira O. L. (2012). Silicon suppresses *Fusarium* wilt development in banana plants. *J. Phytopathol.* 160 674–679 10.1111/jph.12005

[B29] Fox R. L. Silva J. A. Younge O. R. Plucknet D. L. Sherman G. D. (1967). Soil and plant silicon and silicate response by sugarcane. *Soil Sci. Soc. Am. Pro.* 31 775–779 10.2136/sssaj1967.03615995003100060021x

[B30] Galway K. E. Duncan R. P. Syrett P. Emberson R. M. Sheppard A. W. Briese D. T. (2004). “Insect performance and host-plant stress: a review from a biological control perspective,” in *Proceedings of the XI International Symposium on Biological Control of Weeds* ed. Cullen J. M. (Canberra: CSIRO Entomology) 394–399

[B31] Gomes F. B. Moraes J. C. Santos C. D. Goussain M. M. (2005). Resistance induction in wheat plants by silicon and aphids. *Sci. Agric. (Piracicaba, Braz.)* 62 547–551 10.1590/S0103-90162005000600006

[B32] Hanisch H. C. (1981). Die Populationsentwicklung von Getreideblattlausen an Weizenpflanzen nach verschieden hoher Stickstoffdungung und vorbeugender Applikation von Kieselsaure zur Wirtspflanze. *Mitt. Dtsch. Ges. Allg. Angew. Entomol.* 3 308–311

[B33] Hou M.-L. Han Y.-Q. (2010). Silicon-mediated rice plant resistance to the Asiatic rice borer (Lepidoptera: Crambidae): effects of silicon amendment and rice varietal resistance. *J. Econ. Entomol.* 103 1412–1419 10.1603/EC09341 20857756

[B34] Huberty A. F. Denno R. F. (2004). Plant water stress and its consequences for herbivorous insects: a new synthesis. *Ecology* 85 1383–1398 10.1890/03-0352

[B35] Inman-Bamber N. G. (2004). Sugarcane water stress criteria for irrigation and drying off. *Field Crop. Res.* 89 107–122 10.1016/j.fcr.2004.01.018

[B36] Joshi S. V. Zhou M. M. Leslie G. W. Way M. J. Keeping M. G. (2014). Comparison of methods for determining thrips (*Fulmekiola serrata*) damage and implications for resistance screening. *Int. Sugar J.* 116 214–216

[B37] Kanamugire A. Meyer J. H. Haynes R. J. Naidoo G. Keeping M. G. (2006). An assessment of soil extraction methods for predicting the silicon requirement of sugarcane. *Proc. S. Afr. Sug. Technol. Ass.* 80 287–290

[B38] Kanto T. Miyoshi A. Ogawa T. Maekawa K. Aino M. (2006). Suppressive effect of liquid potassium silicate on powdery mildew of strawberry in soil. *J. Gen. Plant Pathol.* 72 137–142 10.1007/s10327-005-0270-8

[B39] Keeping M. Butterfield M. Leslie G. Rutherford S. (2008). Initial measures for management of thrips. *The Link* 17 4–5

[B40] Keeping M. G. (2006). Screening of South African sugarcane cultivars for resistance to the stalk borer, *Eldana saccharina* Walker (Lepidoptera: Pyralidae). *Afr. Entomol.* 14 277–288

[B41] Keeping M. G. Kvedaras O. L. (2008). Silicon as a plant defence against insect herbivory: response to Massey, Ennos and Hartley. *J. Anim. Ecol.* 77 631–633 10.1111/j.1365-2656.2008.01380.x 18341561

[B42] Keeping M. G. Kvedaras O. L. Bruton A. G. (2009). Epidermal silicon in sugarcane: cultivar differences and role in resistance to sugarcane borer *Eldana saccharina*. *Environ. Exp. Bot.* 66 54–60 10.1016/j.envexpbot.2008.12.012

[B43] Keeping M. G. McFarlane S. A. Sewpersad N. Rutherford R. S. (2010). Effects of silicon and plant defence inducers on sugarcane yield parameters, *Eldana saccharina* Walker (Lepidoptera: Pyralidae) and *Fulmekiola serrata* Kobus (Thysanoptera: Thripidae). *Proc. S. Afr. Sug. Technol. Ass.* 83 271–275

[B44] Keeping M. G. Meyer J. H. (2002). Calcium silicate enhances resistance of sugarcane to the African stalk borer *Eldana saccharina* Walker (Lepidoptera: Pyralidae). *Agric. For. Entomol.* 4 265–274 10.1046/j.1461-9563.2002.00150.x

[B45] Keeping M. G. Meyer J. H. (2006). Silicon-mediated resistance of sugarcane to *Eldana saccharina* Walker (Lepidoptera: Pyralidae): effects of silicon source and cultivar. *J. Appl. Entomol.* 130 410–420 10.1111/j.1439-0418.2006.01081.x

[B46] Keeping M. G. Meyer J. H. Sewpersad C. (2013). Soil silicon amendments increase resistance of sugarcane to stalk borer *Eldana saccharina* Walker (Lepidoptera: Pyralidae) under field conditions. *Plant Soil* 363 297–318 10.1007/s11104-012-1325-1

[B47] Keeping M. G. Miles N. Sewpersad C. Sithole S. (2012). Silicon and nitrogen nutrition: effects on stalk borer (*Eldana saccharina*) and sugarcane thrips (*Fulmekiola serrata*). *Proc. S. Afr. Sug. Technol. Ass.* 85 87–90

[B48] Keeping M. G. Rutherford R. S. (2004). Resistance mechanisms of South African sugarcane to the African stalk borer *Eldana saccharina* (Lepidoptera: Pyralidae): a review. *Proc. S. Afr. Sug. Technol. Ass.* 78 307–311

[B49] King A. G. (1989). An assessment of the loss in sugarcane yield caused by the stalk borer, *Eldana saccharina*, in Swaziland. *Proc. S. Afr. Sug. Technol. Ass.* 63 197–201

[B50] Korndörfer A. P. Grisoto E. Vendramin J. D. (2011). Induction of insect plant resistance to the spittlebug *Mahanarva fimbriolata* Stål (Hemiptera: Cercopidae) in sugarcane by silicon application. *Neotrop. Entomol.* 40 387–392 21710035

[B51] Kvedaras O. L. An M. Choi Y. S. Gurr G. M. (2010). Silicon enhances natural enemy attraction and biological control through induced plant defences. *Bull. Entomol. Res.* 100 367–371 10.1017/S0007485309990265 19737442

[B52] Kvedaras O. L. Byrne M. J. Coombes N. E. Keeping M. G. (2009). Influence of plant silicon and sugarcane cultivar on mandibular wear in the stalk borer *Eldana saccharina*. *Agr. For. Entomol.* 11 301–306 10.1111/j.1461-9563.2009.00430.x

[B53] Kvedaras O. L. Keeping M. G. (2007). Silicon impedes stalk penetration by the borer *Eldana saccharina* in sugarcane. *Entomol. Exp. Appl.* 125 103–110 10.1111/j.1570-7458.2007.00604.x

[B54] Kvedaras O. L. Keeping M. G. Goebel F. R. Byrne M. J. (2007a). Larval performance of the pyralid borer *Eldana saccharina* Walker and stalk damage in sugarcane: influence of plant silicon, cultivar and feeding site. *Int. J. Pest Manag.* 53 183–194 10.1080/09670870601110956

[B55] Kvedaras O. L. Keeping M. G. Goebel F. R. Byrne M. J. (2007b). Water stress augments silicon-mediated resistance of susceptible sugarcane cultivars to the stalk borer *Eldana saccharina* (Lepidoptera: Pyralidae). *Bull. Entomol. Res.* 97 175–183 10.1017/S0007485307004853 17411480

[B56] Leslie G. (2004). “Pests of sugarcane,” in *Sugarcane* 2nd Edn ed. James G. (Oxford: Blackwell Science) 78–100 10.1002/9780470995358.ch4

[B57] Leslie G. W. (2009). Estimating the economic injury level and the economic threshold for the use of α-cypermethrin against the sugarcane borer, *Eldana saccharina* Walker (Lepidoptera: Pyralidae). *Int. J. Pest Manag.* 55 37–44 10.1080/09670870802450243

[B58] Leslie G. W. Moodley S. (2011). Developments in the use of insecticides for the control of the sugarcane thrips *Fulmekiola serrata* (Kobus) in South Africa. *Proc. S. Afr. Sug. Technol. Ass.* 84 310–313

[B59] Marafon A. C. Endres L. (2013). Silicon: fertilization and nutrition in higher plants. *Amaz. J. Agric. Environ. Sci.* 56 380–388

[B60] Massey F. P. Ennos A. R. Hartley S. E. (2006). Silica in grasses as a defence against insect herbivores: contrasting effects on folivores and a phloem feeder. *J. Anim. Ecol.* 75 595–603 10.1111/j.1365-2656.2006.01082.x 16638012

[B61] Massey F. P. Hartley S. E. (2009). Physical defences wear you down: progressive and irreversible impacts of silica on insect herbivores. *J. Anim. Ecol.* 78 281–291 10.1111/j.1365-2656.2008.01472.x 18771503

[B62] Mattson W. J. (1980). Herbivory in relation to plant nitrogen content. *Annu. Rev. Ecol. Syst.* 11 119–161 10.1146/annurev.es.11.110180.001003

[B63] Meyer J. H. Harding R. Rampersad A. L. Wood R. A. (1998). Monitoring long term soil fertility trends in the South African sugar industry using the FAS analytical database. *Proc. S. Afr. Sug. Technol. Ass.* 72 61–68

[B64] Meyer J. H. Keeping M. G. (2005). The impact of nitrogen and silicon nutrition on the resistance of sugarcane varieties to *Eldana saccharina* (Lepidoptera: Pyralidae). *Proc. S. Afr. Sug. Technol. Ass.* 79 363–367

[B65] Meyer J. H. Schumann A. W. Wood R. A. Nixon D. J. Berg M. (2007). Recent advances to improve nitrogen use efficiency of sugarcane in the South African sugar industry. *Proc. Int. Soc. Sug. Technol.* 26 238–246

[B66] Miles N. Rhodes R. (2013). *Information Sheet 7.17, Guidelines for the Interpretation of Leaf Analyses for Sugarcane.* Mount Edgecombe: South African Sugarcane Research Institute

[B67] Miles N. van Antwerpen R. van Heerden P. D. R. Rhodes R. Weigel A. McFarlane S. A. (2011). Extractable silicon in soils of the sugar industry and relationships with crop uptake. *Proc. S. Afr. Sug. Technol. Ass.* 84 189–192

[B68] Moberly P. K. Meyer J. H. (1975). The amelioration of acid soils in the South African Sugar Industry. *Fert. Soc. S. Afr. J.* 2 57–66

[B69] Nabity P. D. Orpet R. Miresmailli S. Berenbaum M. R. DeLucia E. H. (2012). Silica and nitrogen modulate physical defense against chewing insect herbivores in bioenergy crops *Miscanthus* × *giganteus* and *Panicum virgatum* (Poaceae). *J. Econ. Entomol.* 105 878–883 10.1603/EC11424 22812125

[B70] Pankhurst C. E. Blair B. L. Magarey R. C. Stirling G. R. Bell M. J. Garside A. L. (2005). Effect of rotation breaks and organic matter amendments on the capacity of soils to develop biological suppression towards soil organisms associated with yield decline of sugarcane. *Appl. Soil Ecol.* 28 271–282 10.1016/j.apsoil.2004.07.010

[B71] Ramburan S. Sewpersad C. McElligott D. (2009). Effects of variety, harvest age and eldana on coastal sugarcane production in South Africa. *Proc. S. Afr. Sug. Technol. Ass.* 82 580–588

[B72] Reynolds O. L. Keeping M. G. Meyer J. H. (2009). Silicon-augmented resistance of plants to herbivorous insects: a review. *Ann. Appl. Biol.* 155 171–186 10.1111/j.1744-7348.2009.00348.x

[B73] Rhodes R. Miles N. Keeping M. G. (2013). Crop nutrition and soil textural effects on eldana damage in sugarcane. *Proc. S. Afr. Sug. Technol. Ass.* 86 212–136

[B74] Rodrigues F. A. Datnoff L. E. Korndörfer G. H. Seebold K. W. Rush M. C. (2001). Effect of silicon and host resistance on sheath blight development in rice. *Plant Dis.* 85 827–832 10.1094/PDIS.2001.85.8.827 30823048

[B75] Romero A. Munévar F. Cayón G. (2011). Silicon and plant diseases. A review. *Agron. Colomb.* 29 473–480

[B76] Rutherford R. S. Conlong D. E. (2010). Combating sugarcane pests in South Africa: from researching biotic interactions to bio-intensive integrated pest management in the field. *Proc. Int. Soc. Sug. Technol.* 27 1–17

[B77] Sétamou M. Schulthess F. Bosque-Pérez N. A. Odjo-Thomas A. (1993). Effect of plant nitrogen and silica on the bionomics of *Sesamia calamistis* (Lepidoptera: Noctuidae). *Bull. Ent. Res.* 83 405–411 10.1017/S000748530002931X

[B78] Shetty R. Jensen B. Shetty N. P. Hansen M. Hansen C. W. Starkey K. R. (2012). Silicon induced resistance against powdery mildew of roses caused by *Podosphaera pannosa*. *Plant Pathol.* 61 120–131 10.1111/j.1365-3059.2011.02493.x

[B79] Stranack R. A. Miles N. (2011). Nitrogen nutrition of sugarcane on an alluvial soil on the Kwazulu-Natal north coast: effects on yield and leaf nutrient concentrations. *Proc. S. Afr. Sug. Technol. Ass.* 84 198–209

[B80] Subramanian S. Gopalaswamy A. (1988). Effect of silicate materials in rice crop pests. *Int. Rice Res. New.* 13 32

[B81] Thorburn P. J. Probert M. E. Lisson S. Wood A. W. Keating B. A. (1999). Impacts of trash retention on soil nitrogen and water: an example from the Australian sugarcane industry. *Proc. S. Afr. Sug. Technol. Ass.* 75–79

[B82] Van Antwerpen R. Conlong D. E. Miles N. (2011). Nutrient management options for reducing *Eldana saccharina* (Lepidoptera: Pyralidae) infestation of trashed sugarcane fields: results from a preliminary study. *Proc. S. Afr. Sug. Technol. Ass.* 84 298–300

[B83] Van Der Laan M. Miles N. (2010). Nutrition of the South African sugar crop: current status and long-term trends. *Proc. S. Afr. Sug. Technol. Ass.* 83 195–204

[B84] Van Dillewijn C. (1952). *Botany of Sugarcane*. Waltham, MA: The Chronica Botanica Co.: Book Department

[B85] Way M. Keeping M. Leslie G. (2007). Update on thrips. *The Link* 16:10

[B86] Way M. J. (1995). Developmental biology of the immature stages of *Eldana saccharina* Walker (Lepidoptera: Pyralidae). *Proc. S. Afr. Sug. Technol. Ass.* 69 83–86

[B87] Way M. J. Leslie G. W. Keeping M. G. Govender A. (2006a). Incidence of *Fulmekiola serrata* (Thysanoptera: Thripidae) in South African sugarcane. *Proc. S. Afr. Sug. Technol. Ass.* 80 199–201

[B88] Way M. J. Stiller M. Leslie G. W. Conlong D. E. Keeping M. G. Rutherford R. S. (2006b). *Fulmekiola serrata* (Kobus) (Thysanoptera: Thripidae), a new pest in southern African sugarcane. *Afr. Entomol.* 14 401–403

[B89] Way M. J. Rutherford R. S. Sewpersad C. Leslie G. W. Keeping M. G. (2010). Impact of sugarcane thrips, *Fulmekiola serrata* (Kobus) (Thysanoptera: Thripidae) on sugarcane yield in field trials. *Proc. S. Afr. Sug. Technol. Ass.* 83 244–256

[B90] White T. C. R. (1984). The abundance of invertebrate herbivores in relation to the availability of nitrogen in stressed food plants. *Oecologia* 63 90–105 10.1007/BF00379790 28311171

[B91] Williams J. R. (1956). Varietal susceptibility in sugarcane to the thrips *Fulmekiola serrata* (Kob.). *Proc. Int. Soc. Sug. Technol.* 9 789–799

[B92] Ye M. Song Y. Y. Long J. Wang R. L. Baerson S. R. Pan Z. Q. (2013). Priming of jasmonate-mediated antiherbivore defense responses in rice by silicon. *Proc. Natl. Acad. Sci. U.S.A*. 110 E3631–E3639 10.1073/pnas.1305848110 24003150 PMC3780902

